# Similarity to Self-Antigens Shapes Epitope Recognition from Viruses Under Autoimmune and Infectious Disease

**DOI:** 10.3390/ijms26136041

**Published:** 2025-06-24

**Authors:** Alvaro Ras-Carmona, Alexander Lehmann, Pedro A. Reche

**Affiliations:** Department of Immunology, Ophthalmology and ENT, Faculty of Medicine, University Complutense of Madrid, Pza Ramon y Cajal S/N, 28040 Madrid, Spain; aras@ucm.es (A.R.-C.); allehman@ucm.es (A.L.)

**Keywords:** molecular mimicry, cross-reactivity, epitope, viruses, autoimmunity, infectious disease

## Abstract

Self/non-self-discrimination is a fundamental aspect of adaptive immunity, which helps prevent harmful autoimmune responses. However, infectious agents can also act as environmental catalysts for autoimmune diseases. In this study, we investigated the role of molecular mimicry to self-antigens in epitope recognition in relation to infectious and autoimmune diseases. To this end, we performed BLAST searches against the human proteome, utilizing known virus-specific B and T cell peptide epitopes identified in association with autoimmune or infectious diseases in humans as our queries. Additionally, similar control analyses were carried out using non-B and non-T cell epitopes, consisting of random viral peptide sequences. Overall, our results endorsed a major role of molecular mimicry in instigating or sustaining autoimmunity associated with viral infections and challenged the prevailing view on self/non-self-discrimination for T cells. Additionally, we uncovered many virus-specific epitopes among those identified in association with infectious diseases with high similarity to self-antigens, which are primarily derived from human coronaviruses and various flaviviruses. Recognition of these epitopes could lead to autoimmunity against human proteins that are in cellular components concerning cell motility, cell membrane projections, and cellular synapses.

## 1. Introduction

Adaptive immunity is mediated by T and B cells, which recognize antigens through specific receptors, known as the T cell receptor (TCR) and the B cell receptor (BCR), respectively [1]. More specifically, B and T cells recognize small regions within their cognate antigens, known as epitopes. B cell epitopes, which are also the targets of antibodies, can be recognized on intact antigens and must be solvent-accessible. In contrast, T cells recognize epitope peptides from antigen degradation, which must be present on the surface of antigen-presenting cells, bound to major histocompatibility complex (MHC) molecules, referred to as human leukocyte antigen (HLA) molecules [1] in humans. B and T cell immune responses are specific thanks to the presence of a large repertoire of B and T cells expressing distinct antigen receptors.

BCR and TCR diversity is generated during B and T cell development, which produces a vast repertoire of B and T cells, each with distinct antigen recognition. Moreover, cells with self-reactive antigen receptors are deleted through a process known as negative selection [2,3,4]. This process provides a mechanism of central tolerance, which is key to distinguishing foreign from self-antigens and avoiding misguided immune responses [3,4]. However, central tolerance does not eliminate all autoreactive B and T cells. As a result, additional mechanisms of peripheral tolerance exist to avoid harmful autoimmune responses [5,6,7], such as those mediated by T regulatory cells [8].

Despite the many immune regulatory controls, immune tolerance can break down and lead to autoimmunity. Indeed, autoimmune diseases are common, affecting an estimated 10% of the population, with their prevalence expected to continue rising worldwide [9,10]. Clinical symptoms of autoimmune diseases vary greatly, depending on the targeted self-antigens, their localization in the body, and the mechanism of the autoimmune response [11]. Determining the cause of autoimmune diseases is challenging because of their multifactorial nature, involving both genetic and environmental factors [11]. In general, HLA gene alleles show the strongest association with autoimmune diseases [12,13]. Known environmental factors that can trigger/influence autoimmunity include nutrients, exposure to chemicals and medications, and infections [14,15].

Infectious diseases are a major factor contributing to the development of autoimmune conditions [16,17]. While various classes of pathogens (parasites, bacteria, and viruses) have been associated with the initiation and progression of autoimmune disorders, viruses have received special attention [18,19]. In fact, in animal models, viral infections have been shown to induce autoimmunity [20]. In humans, several viruses have been commonly linked with autoimmune disorders, including members of the Herpesvirus family, Coxsackie-B-virus, and Rubella virus [19]. However, many other viruses have been associated with autoimmune diseases [19] and, to our knowledge, a complete catalogue of viruses linked to autoimmune diseases has yet to be established.

An important mechanism by which viral infections—and infectious agents, in general—may induce autoimmunity is molecular mimicry [21,22,23]. It occurs when T or B cells become activated against epitopes in foreign antigens that are similar to epitopes in self-antigens, leading to a cross-reactive autoimmune response in susceptible individuals. However, molecular mimicry is not the only mechanism by which infections can lead to breaches in immune tolerance and autoimmunity. Non-specific bystander activation, or persistent antigenic stimuli, may also contribute to the development of autoimmune diseases [21]. Moreover, infection can also promote epitope spreading, a mechanism that diversifies the response of autoreactive cells induced by infection so as to target additional self-epitopes [24]. Therefore, the overall contribution of molecular mimicry to the development of autoimmunity is unknown. Similarly, the extent of molecular mimicry/similarity among epitopes and self-antigens that are targeted during infections, in or outside of the context of autoimmune diseases, has yet to be investigated.

In this study, we examined molecular mimicry/similarity to self-antigens of known virus-specific linear B and T cell epitopes identified in association with autoimmune or infectious diseases in humans. Utilizing BLAST searches, we compared the similarity of these viral epitopes to the human proteome. We also analyzed the similarity to self-antigens of non-epitopes, consisting of random peptides obtained from the viruses linked to autoimmune diseases. As a result, we found that virus-specific B and T cell epitopes verified in the context of autoimmune diseases are significantly more similar to self-antigens than those identified outside of an autoimmune context, associated with infectious diseases. Interestingly, we also found that the similarity to self-antigens of T cell epitopes is enhanced with regard to non-epitopes, challenging the common view on self/non-self-discrimination for T cells. Moreover, by investigating virus-specific epitopes associated with infectious diseases, without a known link to autoimmunity, and high similarity to self-antigens, we uncovered potential sources of autoimmunity due to molecular mimicry.

## 2. Results

### 2.1. Molecular Mimicry Between Viral Epitopes and the Human Proteome

**Figure 1:**
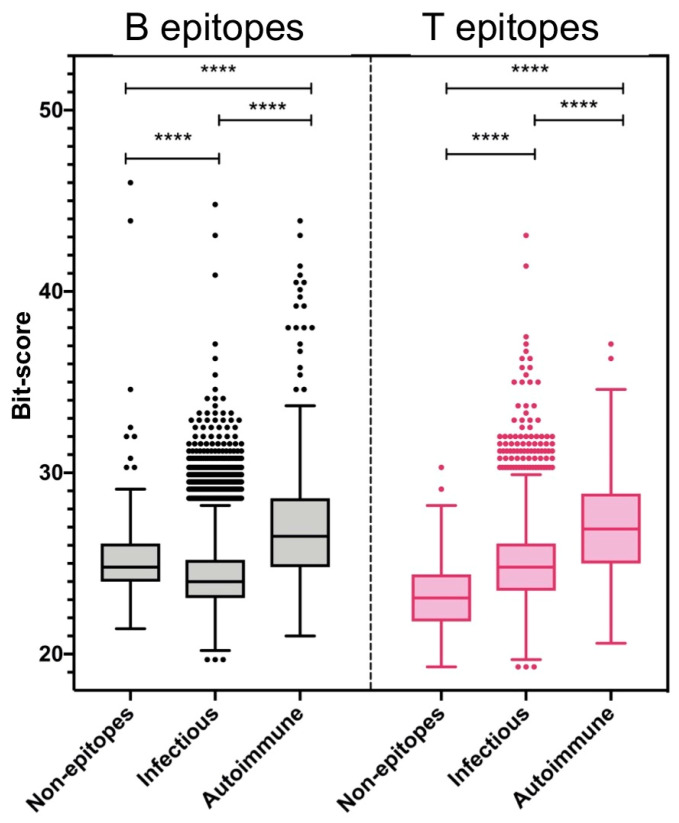
Sequence similarity between virus-specific B and T cell epitopes and self-antigens. Box-and-whisker plots showing similarity to self-antigens as judged by bit-scores (Y-axis) of virus-specific B (gray) and T cell epitopes (pink) linked to infectious and autoimmune diseases (X-axis). Bit-scores of non-epitopes to self-antigens are also plotted. Non-epitopes consist of random peptides of the same length and viral source as the B and T cell epitopes linked to autoimmune diseases. The boxes illustrate the first (Q1) and third (Q3) quartiles of the data (bit-scores) and the median value, while the whiskers indicate the maximum and minimum data values, excluding outliers. In the plot, the maximum whisker is calculated as follows: Q3 + (Q3 − Q1) × 1.5, whereas the minimum whisker is determined by the formula: Q1 − (Q3 − Q1) × 1.5. It is noteworthy that the bit-scores of viral epitopes associated with infectious diseases seem to be less homogeneous than those of viral epitopes associated with autoimmune diseases, comprising a greater number of high-similarity outliers. Bit-scores were obtained after BLASTP searches against the human proteome using, as a query, the relevant epitope sequences and considering only the maximum bit-score *per* query sequence (details in the Materials and Methods). Statistically significant differences between the different sets of B and T cell epitopes and corresponding random peptides were determined by Mann–Whitney U tests. **** *p*-value < 0.0001.

We used sequence similarity to investigate the role of molecular mimicry in determining epitope recognition from viruses in the context of autoimmune and infectious diseases. To that end, we used virus-specific T and B cell epitopes available in the Immune Epitope Database (IEDB) [25,26] that have been reported as recognized by humans in two disease contexts: autoimmune and infectious diseases (details in the Materials and Methods). The IEDB collects epitopes and related data from peer-reviewed publications. Accordingly, the viral epitopes identified in the context of autoimmune diseases in humans consist of viral epitopes detected in humans suffering from autoimmune diseases generally associated with the relevant viruses. In contrast, should epitopes from the same viruses be identified in humans who do not have autoimmune diseases, these epitopes are classified in the IEDB as associated with infectious diseases. Likewise, any viral epitope identified in humans suffering from infections, outside of an autoimmune context, is classified in the IEDB as linked with infectious diseases. As B cell epitopes, we only considered linear B cell epitope peptides, which represent the vast majority of virus-specific B cell epitopes in the IEDB [27]. We specifically assembled two independent non-overlapping datasets of virus-specific B cell epitopes, one encompassing 196 B cell epitopes linked to autoimmune diseases (Supplementary Dataset S1) and the other encompassing 30488 B cell epitopes linked to infectious diseases (Supplementary Dataset S2). Likewise, we collected virus-specific T cell epitopes in two independent non-overlapping datasets: one consisting of 241 T cell epitopes determined in association with autoimmune diseases (Supplementary Dataset S3) and the other of 5292 T cell epitopes determined in association with infectious diseases (Supplementary Dataset S4). In addition to the sequence of the epitopes, the datasets included data related to the source of the epitopes (virus and antigen), and, in the case of T cell epitopes, also included the HLA molecules restricting the T cell epitopes. Subsequently, we carried out BLAST searches with the epitope sequences against the human proteome, using BLAST bit-scores as a measure of sequence similarity/mimicry to self-antigens. Only the maximum bit-score per sequence was considered. The same similarity analysis was also applied to non-epitopes, consisting of random peptide sequences with the same length as B cell and T cell epitopes linked to autoimmunity that were obtained from the corresponding viruses (provided in Supplementary Datasets S5 and S6). The resulting bit-scores of virus-specific B and T cell epitopes linked to autoimmune or infectious diseases, as well as those of random peptides, are shown in Figure 1 and are included in the mentioned Supplementary Datasets. According to the bit-scores, B cell epitopes from viruses linked to autoimmune diseases are significantly more similar to self-antigens than B cell epitopes linked to infectious diseases. The median bit-score value of B cell epitopes linked to autoimmunity is 26.50, while the median bit-score value of B cell epitopes linked to infectious diseases is 24.00. This difference is statistically significant, as judged by Mann–Whitney U Tests (*p* < 0.001). B cell epitopes linked to autoimmune diseases are also significantly more similar to self-antigens than non-epitopes (median bit-score = 24.89) (*p* ≤ 0.001), while B cell epitopes linked to infectious diseases are significantly less similar to self-antigens than non-epitopes (*p* ≤ 0.001) (Figure 1). With regard to the T cell epitopes, those linked to autoimmune diseases are also more similar to self-antigens (median bit-score = 26.90) than those linked to infectious diseases (median bit-score = 24.80), and the difference is also statistically significant (*p* < 0.001). Interestingly, unlike B cell epitopes, virus-specific T cell epitopes linked to infectious diseases are significantly more similar to self-antigens than non-epitopes (median bit-score = 23.19) (Figure 1). As will be discussed, these results suggest that molecular mimicry/similarity between epitopes in foreign antigens and self-antigens could play a major role in initiating and/or maintaining autoimmune diseases and challenge the common view on self/non-self-discrimination for T cells.

It is noteworthy that the bit-scores of epitopes identified in the context of infectious diseases seem to be less homogeneous than those of viral epitopes identified in the context of autoimmunity, comprising a greater number of high-similarity outliers (Figure 1). The vast majority of viral epitopes identified in the context of infectious diseases have bit-scores under the noted median values of viral epitopes identified in the context of autoimmune diseases. Thus, 93.0% of B cell epitopes linked with infectious diseases have a bit-score under 26.50 (median in autoimmunity), while 87.6% of T cell epitopes linked with infectious diseases have a bit-score under 26.90 (median in autoimmunity). Interestingly, although being a minority, there are substantial virus-specific B and T cell epitopes identified in the context of infectious diseases, with a similarity to self-antigens above the mimicry threshold defined by the median values in autoimmunity. Specifically, 2122 B cell epitopes and 653 T cell epitopes have bit-scores above the median bit-score of B and T cell epitopes linked to autoimmune diseases, 26.50 and 26.90, respectively (provided in Supplementary Dataset S7). Subsequently, we explored the potential connection of these B and T cell epitopes with autoimmunity due to molecular mimicry.

### 2.2. HLA Restriction of T Cell Epitopes Linked to Infectious Diseases with High Similarity to Human Antigens

**Figure 2:**
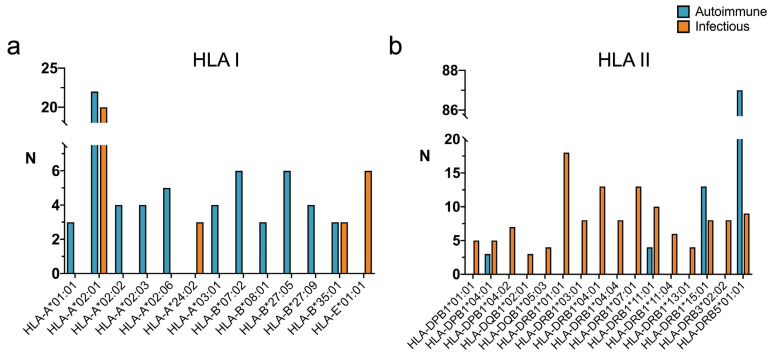
HLA restriction of virus-specific T cell epitopes linked to autoimmune diseases or to infectious diseases with high similarity to self-antigens. Bar plots depicting HLA I (panel **a**) and HLA II (panel **b**) molecules restricting T cell epitopes linked with autoimmune (blue) or infectious diseases (orange). Selected T cell epitopes linked to infectious diseases have a similarity to self-antigens, as judged by bit-scores, above the median value of T cell epitopes linked with autoimmune diseases. Only HLA alleles found to restrict more than 3 T cell epitopes are depicted.

Given the well-established association between autoimmunity and HLA, we compared the HLA restriction of virus-specific T cell epitopes linked to infectious diseases and highly similar to self-antigens with that of virus-specific T cell epitopes linked to autoimmunity. In this analysis, we only considered T cell epitopes with HLA restriction elements known at the gene allele level and distinguished between HLA I- and HLA II-restricted T cell epitopes. In Figure 2, we show the identity of HLA I and HLA II elements restricting at least three virus-specific T cell epitopes. Overall, the majority of virus-specific T cell epitopes linked to autoimmune diseases are restricted by HLA II molecules (102 T cell epitopes out of 140) and, likewise, virus-specific T cell epitopes linked to infectious diseases with high similarity to self-antigens (135 T cell epitopes out of 178). HLA-A*02:01 is the most common allele among HLA I-restricted virus-specific T cell epitopes linked to either autoimmune or infectious diseases (Figure 2a). Meanwhile, HLA-DRB1*01:01 is the most common allele among HLA II-restricted virus-specific T cell epitopes linked to infectious diseases, and HLA-DRB5*01:01 is the most common one linked to autoimmune diseases (Figure 2b). Moreover, all HLA II alleles restricting T cell epitopes linked to autoimmune diseases are also found to restrict T cell epitopes linked to infectious diseases with high similarity to self-antigens.

### 2.3. Viruses with Epitopes Linked to Infectious Diseases with High Similarity to Self-Antigens

**Figure 3:**
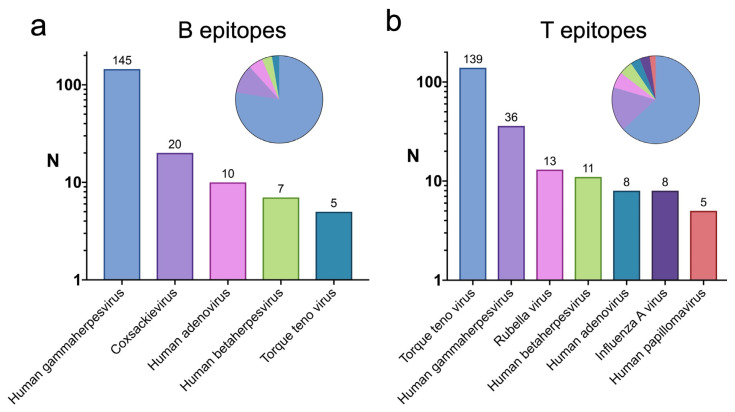
Viruses bearing B and T cell epitopes linked with autoimmune diseases. Bar plots showing the viruses with B and T cell epitopes linked to autoimmune diseases are shown in panels (**a**,**b**), respectively. The number of epitopes is plotted on a log10 scale (Y-axis), and only those viruses bearing more than 5 distinct epitopes are shown (X-axis). Pie charts on top of bar plots depict the proportion of epitopes in the noted viruses, considering only those bearing more than 5 epitopes.

We first identified the viruses bearing B and T cell epitopes reported in the context of autoimmune diseases. B cell epitopes linked to autoimmune diseases are present mostly on various Human herpesviruses, Coxsackievirus, Human adenoviruses, and Torque teno viruses (Figure 3a). These same viruses, except Coxsackievirus, are also significant sources of virus-specific T cell epitopes identified in the context of autoimmune diseases (Figure 3b). Other significant viruses bearing T cell epitopes linked to autoimmune diseases are Rubella virus, Influenza A virus, and Human papillomavirus (Figure 3b)

**Figure 4:**
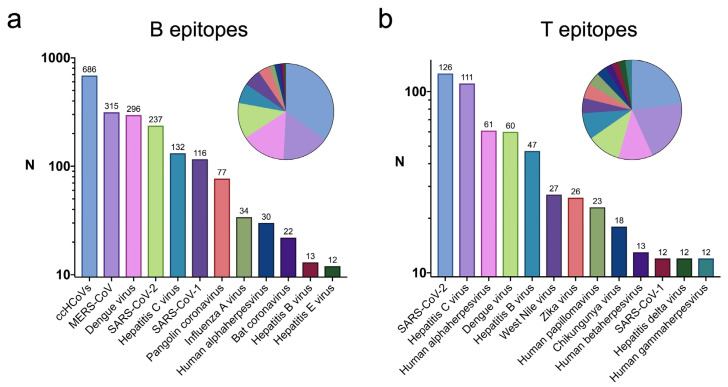
Viruses with B and T cell epitopes linked to infectious diseases with high similarity to self-antigens. Bar plots showing the number of B (**a**) and T (**b**) cell epitopes linked to infectious diseases with high similarity to self-antigens that are present in different viruses. The number of epitopes is shown on a log10 scale (X-axis), and only viruses (Y-axis) bearing more than ten epitopes are represented.

Next, we identified the viruses encompassing B and T cell epitopes linked to infectious diseases with high similarity to self-antigens. In Figure 4, we show the identified viruses bearing more than 10 of these epitopes. With regard to B cell epitopes, 1476 out of 2122 virus-specific B cell epitopes with high similarity to self-antigens linked to infectious diseases are present in different coronaviruses, including common cold human coronavirus (299E, NL63, HKU1, and OC43) (686 epitopes), SARS-CoV-1 (116 epitopes), SARS-CoV-2 (237 epitopes), and MERS-CoV (315 epitopes) (Figure 4a). Other relevant sources of B cell epitopes with high similarity to self-antigens are Dengue virus (296 epitopes), Hepatitis C virus (132 epitopes), Influenza A virus (34 epitopes), Human alphaherpesvirus (30 epitopes), and Hepatitis B virus (13 epitopes). T cell epitopes linked to infectious diseases with high similarity to self-antigens have similar viral sources to B cell epitopes. Thus, many of these T cell epitopes are found in SARS-CoV-2 (126 epitopes), Hepatitis C virus (111 epitopes), Human alphaherpesvirus (61 epitopes), Dengue virus (60 epitopes), and Hepatitis B virus (47 epitopes) (Figure 4b). In addition, they are also present in West Nile virus (27 epitopes), Zika virus (26 epitopes), Human papillomavirus (23 epitopes), Chikungunya virus (18 epitopes), Human betaherpesviruses (13 epitopes), and Hepatitis delta virus (12 epitopes). However, no B cell epitopes with high similarity to self-antigens are present in these latter viruses.

### 2.4. Targets of Potential Cross-Reactive Autoimmunity Due to Molecular Mimicry

**Figure 5:**
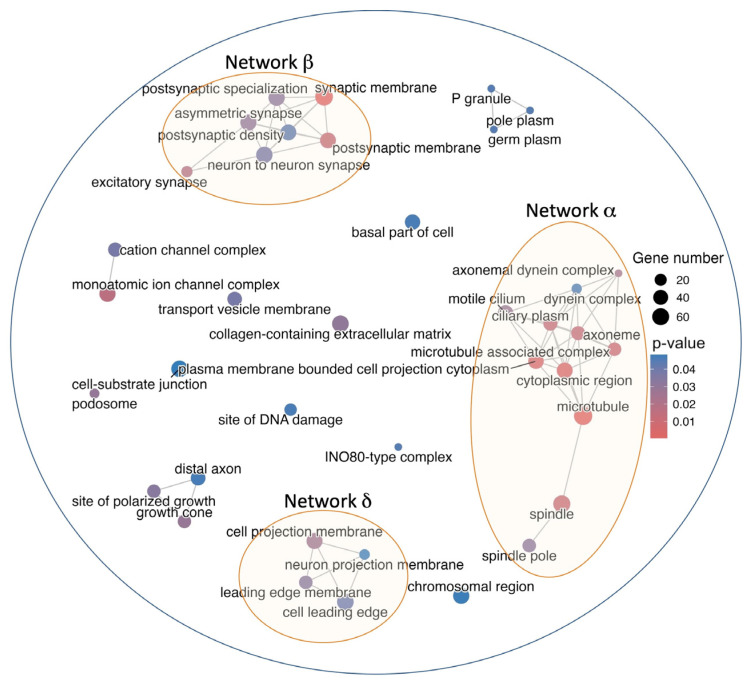
Cellular components of human proteins targeted by virus-specific epitopes. Network map showing the results of the Gene Ontology (GO) enrichment analysis based on cellular component terms/annotations of human proteins with high similarity to virus-specific epitopes linked to infectious diseases. Cellular components are represented by bubbles and are connected by lines when sharing common genes. Three major networks are labeled as α, β, and δ. The bubble size is proportional to the number of genes/proteins, and the color is linked to the corrected *p*-value, as indicated by the colored bar.

Virus-specific epitopes linked to infectious diseases with high similarity to self-antigens (2122 B and 653 T cell epitopes) targeted 1761 distinct human proteins, and we investigated their potential implication in autoimmunity. To that end, we carried out a Gene Ontology (GO) enrichment analysis using cellular component terms/annotations of the genes encoding the targeted human proteins (details in the Materials and Methods). As a result, we obtained 38 GO cellular components that were enriched in the targeted proteins. These cellular components are visualized in Figure 5 as a network map, where cellular components with common genes are connected. Most cellular components share common genes, and we observed the presence of three major networks, which we noted as α, β, and δ, connecting more than three enriched cellular components (Figure 5). Network α encompasses genes with enriched cellular components connected to microtubules and motility; Network β includes genes with enriched cellular components related to membrane synapses; and Network δ includes enriched cellular components connected to membrane projections.

**Figure 6:**
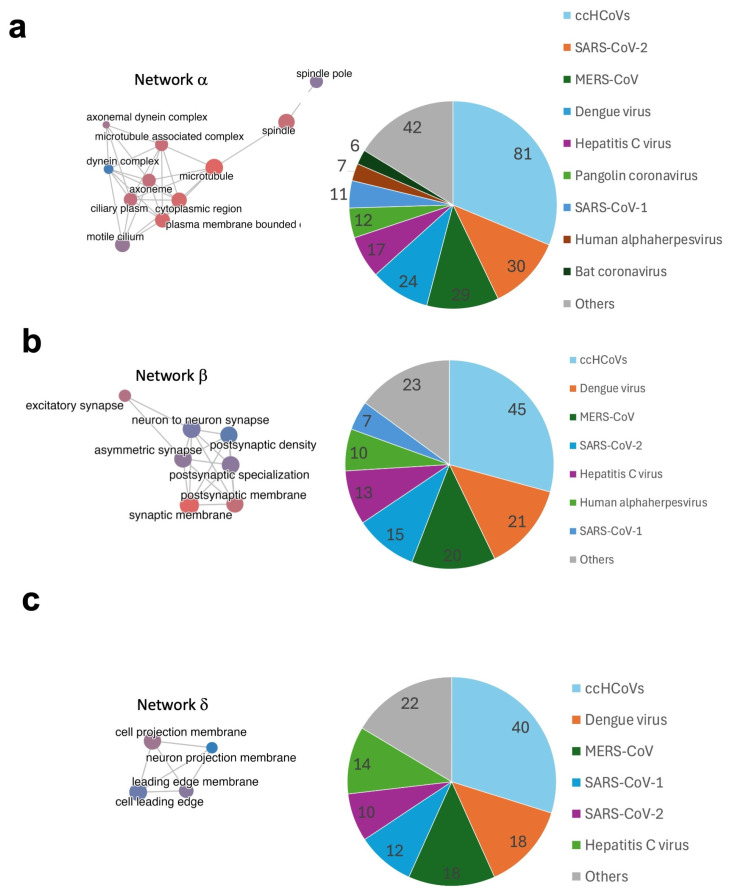
Epitope virus contribution to major networks of cellular components. Pie charts showing the epitope contribution of viruses linked to infectious diseases to selected networks of enriched cellular components depicted by the targeted human proteins. (**a**) Microtubule-motility network; (**b**) synaptic membrane network; (**c**) projection membrane network.

Given the relevance and extent of the noted networks, we identified the epitope contribution from viruses targeting the proteins/genes included in the relevant cellular components. As shown in Figure 6a–c, the major epitope contributions for Networks α, β and δ are from ccHCoVs. Moreover, coronaviruses, in general, make a relevant contribution to all noted networks. After ccHCoVs, the viruses with the largest epitope contribution varied. Dengue virus is the second major contributor to Networks β and δ, which include genes in enriched cellular components related to membrane synapses and membrane projections, respectively, while SARS-CoV-2 is the second major contributor to Network α.

## 3. Discussion

**Figure 7:**
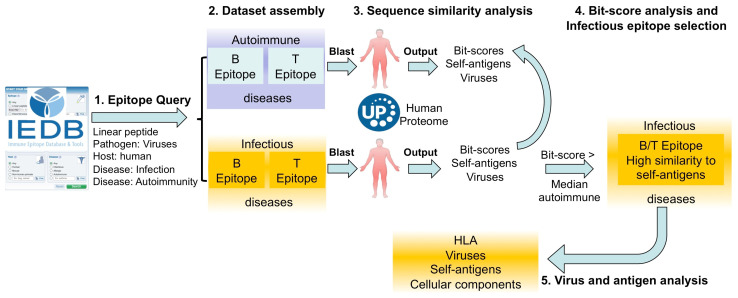
Schematic workflow. Flowchart illustrating the primary steps involved in the analysis of molecular mimicry conducted at the sequence level using experimentally identified linear B and T cell peptide epitopes available in the IEDB. Additionally, control analyses for comparative purposes were carried out using non-B and non-T cell epitopes, which comprised random viral peptide sequences.

Certain viral infections are known environmental triggers of autoimmune diseases, in which B and/or T cells wrongly respond against self-antigens. A potential mechanism contributing to the development of autoimmune conditions linked to infectious agents is molecular mimicry, where sequence and/or structural similarities between epitopes in foreign antigens and their counterparts in self-antigens result in the activation of autoreactive T or B cells. However, infectious agents can disrupt immune tolerance and favor autoimmune diseases through different mechanisms, and the overall role and contribution of molecular mimicry are unknown. In this work, we addressed this issue by using BLAST searches to investigate the sequence similarity to the human proteome of known virus-specific linear B and T cell epitopes available in the immune epitope database (IEDB) that have been identified in connection with infectious or autoimmune diseases in humans, as depicted in Figure 7. In addition, we analyzed the similarity to self-antigens of non-epitopes, consisting of random peptides obtained from viruses. It is worth pointing out that our approach can only detect molecular mimicry at the sequence level, and, therefore, we only considered linear B cell epitopes, which, incidentally, are the majority in the IEDB [27]. However, unlike T cell epitopes, B cell epitopes can also be conformational, which are arguably the most relevant B cell epitopes, as they are readily accessible for antibody recognition in the tertiary structure of the native antigen. Therefore, although a minority in the IEDB, our study could have been enhanced by examining the molecular mimicry of conformational B cell epitopes or by identifying the structural similarity of linear B cell epitopes to the tertiary structure of human proteins, following an approach similar to that reported by Balbin et al. [28].

Regardless of the mentioned shortcomings, our results clearly show that virus-specific B and T cell epitopes linked to autoimmune diseases are, overall, significantly more similar to self-antigens than those linked to infectious diseases (Figure 1). This finding suggests that molecular mimicry can indeed represent a major mechanism behind autoimmune diseases linked to viral infections. However, whether similarity/mimicry between viral epitopes and self-antigens triggers or just sustains autoimmunity remains unclear. Indeed, the loss of immune tolerance by some other mechanisms could potentially facilitate the recognition of viral epitopes that are more similar to self-antigens. Nonetheless, virus-specific epitopes linked to autoimmune diseases are also more similar to self-antigens than non-epitopes, which may suggest a potential triggering role (Figure 1). Interestingly, virus-specific B and T cell epitopes linked to infectious diseases differ when compared to non-epitopes with regard to their similarity to self-antigens.

B cell epitopes linked to infectious diseases are significantly less similar to self-antigens than non-epitopes (Figure 1). This observation is consistent with the general view on self/non-self-discrimination. According to this view, adaptive immune responses are directed towards epitopes in foreign antigens with little or no similarity to those in self-antigens as a result of negative selection. This process eliminates autoreactive B and T cell clones that, during development, acquire antigen receptors recognizing self-antigens [2,3,4]. In contrast, virus-specific T cell epitopes linked to infectious diseases are more similar to self-antigens than non-epitopes (Figure 1). This result challenges the common self/non-self-discrimination view for T cells, as well as interpretations correlating T cell epitope immunogenicity and dissimilarity to self-peptides [29,30,31]. Moreover, it aligns with the work of Koncz et al. [32], who also found that dissimilar peptides are less likely to be recognized by T cells. As anticipated by Koncz et al. [32], the lack of recognition of dissimilar peptides is connected to the process of positive selection. In addition to negative selection, T cells undergo a positive selection process during maturation, which guarantees that T cells acquire TCRs capable of recognizing host MHC molecules with bound self-peptides [33,34,35]. As a result, surviving T cells are prone to recognize foreign peptides that are similar to the self-peptides recognized during positive selection. Therefore, sequence similarity to self-antigens can be a favorable rather than a detrimental consideration for predicting T cell epitope immunogenicity. However, within certain boundaries, too much similarity to self-antigens may also dampen immunogenicity [36] or, worse, enhance the chance of autoimmune responses. Based on our results, lower and upper limits of T cell epitope similarity to self-antigens could be set to select the most appropriate T cell epitopes from viruses. For example, selecting T cell epitopes with a similarity bit-score to self-antigens above the median of non-epitopes (bit-score > 23.19) and below the median of T cell epitopes linked to autoimmune diseases (bit-score < 26.90) would be appropriate.

As a result of this study, we noted that a considerable number of virus-specific B and T cell epitopes identified in the context of infectious diseases show sequence similarity to self-antigens greater than the median of those identified in the context of autoimmune diseases (Figure 1). These epitopes included 2122 linear B cell epitopes and 653 T cell epitopes. According to the IEDB, none of these epitopes have been identified in subjects afflicted with autoimmune diseases; in fact, the sets of epitopes linked with autoimmune and infectious diseases do not overlap. Nevertheless, we investigated them as potential drivers of autoimmunity due to molecular mimicry. Autoimmune diseases are often associated with particular HLA molecules, representing major susceptibility factors [12,13]. Interestingly, there is a considerable overlap between the human leukocyte antigen (HLA) alleles restricting the T cell epitopes linked to infectious diseases with high similarity to self-antigens and those linked to autoimmune diseases (Figure 2). All HLA II molecules restricting epitopes associated with autoimmune diseases are represented among those of the epitopes linked to infectious diseases with high similarity to self-antigens. The shared HLA II molecules include HLA-DPB1*04:01, HLA-DRB1*11:01, HLA-DRB1*15:01, and HLA-DRB5*01:01 (Figure 2b), while the shared HLA I molecules include HLA-A*02:01 and HLA-B*35:01 (Figure 2a). Many virus-specific T cell epitopes linked to infectious diseases with high similarity to self-antigens are restricted by HLA molecules that are not among those restricting T cell epitopes linked to autoimmune diseases, perhaps representing novel HLA susceptibility factors of autoimmune diseases linked to infections by the relevant viruses.

Virus-specific B and T cell epitopes identified in the context of autoimmune diseases were found in common suspects associated with autoimmunity, including Human herpesviruses, Coxsackievirus, Human adenoviruses, and Rubella virus, as well as less known suspects like Torque teno virus (TTV), Influenza A virus (IAV), and Human papilloma virus (HPV) (Figure 3a,b). Nonetheless, there are reports linking TTV, IAV, and HPV to autoimmunity [37,38,39,40,41,42]. Some virus-specific epitopes linked to infectious diseases with high similarity to self-antigens were also found in the same viruses as those linked to autoimmunity, including IAV, HPV, and Human herpesviruses (Figure 4a,b), which would support a potential implication of these epitopes in triggering cross-reactive autoimmunity. However, virus-specific epitopes linked to infectious diseases with high similarity to self-antigens were mostly found in coronaviruses, including common coronaviruses (ccHCoVs), MERS-CoV, and SARS-CoV-1 and -2, in various members of the flaviviridae family, like Dengue virus (DENV), Hepatitis C virus (HCV), West Nile virus (WNV), and Zika virus (ZKV), and in Hepatitis B virus (HBV) (Figure 4a,b). Among the non-coronaviruses, only HCV has reported epitopes linked to autoimmunity in the IEDB. However, we found evidence of an association between all of them (DENV, HCV, WNV, HBV, and ZKV) and certain autoimmune conditions [43,44,45,46,47]. Hence, recognition of the selected epitopes from these viruses with high similarity to self-antigens could indeed be implicated in triggering autoreactive immunity. Regarding the coronaviruses, none of them have reported epitopes linked to autoimmunity. Still, there are clinical clues connecting the onset of autoimmune diseases following SARS-CoV-2 infection [48,49], and the recognition of the noted epitopes with high similarity to self-antigens may have a triggering role. Potential autoimmunity resulting from molecular mimicry at the tertiary structure between SARS-CoV-2 and human proteins has also been reported [50]. Furthermore, it has been observed that monoclonal antibodies against SARS-CoV-2 exhibit cross-reactivity with human tissue antigens, highlighting the potential risk for autoimmunity and multi-system disorders associated with this virus [51]. In contrast, we could not find clinical reports connecting autoimmunity to ccHCoVs, MERS-CoV, or SARS-CoV-1. However, the molecular mimicry detected in this study, along with structural and clinical similarities to SARS-CoV-2, suggests that a link between these viruses and the onset of autoimmunity in susceptible subjects is plausible. Why such associations have not been observed can be a matter of speculation. MERS-CoV and SARS-CoV-1 infections have had little reach in the population, which hinders our ability to make clinical connections to autoimmune diseases. In contrast, ccHCoV infections are very common [52,53], and it cannot be discounted that their association with autoimmune disease may have been neglected, passed unnoticed, or attributed to concomitant infections. Alternatively, the absence of such an association might suggest that, regardless of the extent of molecular mimicry, some viral infections can rarely promote autoimmune diseases.

Autoimmune manifestations depend greatly on the targeted self-antigens. In this regard, virus-specific epitopes linked to infectious diseases with high similarity to self-antigens targeted 1761 distinct human proteins (Supplementary File S7). Interestingly, we confirmed that the vast majority of these human proteins (1700 out of 1761) are included in the human autoantigen database (AAgAtlas database) available at http://biokb.ncpsb.org.cn/aagatlas_portal/index.php (accessed on 15 June 2025) [54]. The AAgAtlas database includes 8045 human autoantigens that are known targets of autoantibodies. However, there could be additional autoantigens targeted only by T cells. To understand the consequences of targeting these 1761 proteins with high similarity to viral epitopes identified in the context of infectious diseases would require a one-by-one analysis. However, overall, the potential autoimmune disturbances are profound, as revealed by a GO enrichment analysis based on cellular component annotations (Figure 5). According to this analysis, many of the targeted proteins lie in interconnected cellular components (noted as Networks α, β, and δ) related to microtubules and motility (Network α), cellular membrane synapses (Network β), and cell membrane projections (Network δ). Some cellular components in Networks β and δ involve neurons, and we can foresee neurological disturbances resulting from autoimmunity triggered by the recognition of the selected viral epitopes with molecular mimicry to self-antigens. Dengue virus and SARS-CoV-2, which are two major viruses contributing with epitopes to Networks β and δ (Figure 6b,c), are indeed known to induce neurological complications [55,56]. Neurological disturbances also manifest in long or persistent COVID [57,58,59], which, according to our data, could be an autoimmune condition triggered by SARS-CoV-2 infection. Other viruses, such as Hepatitis C virus, that contribute to Networks β and δ are also known to produce neurological disturbances [60], which could be due to autoreactive immune responses triggered by molecular mimicry. The specific consequences of autoimmune responses targeting the proteins belonging to the cellular components concerning microtubules and cell motility (Network α) are hard to pinpoint but could be multiple and profound since they are ubiquitous. SARS-CoV-2 is the major source of epitopes targeting the proteins in this network of cellular components, suggesting that the many ailments observed in susceptible subjects after SARS-CoV-2 infection, particularly those associated with long COVID, are the consequences of autoimmunity.

## 4. Materials and Methods

### 4.1. Epitope Data Collection and Processing

B and T cell linear peptide epitopes were obtained from the Immune Epitope Database (IEDB) [25,26] after independent searches with the following settings: epitope: linear peptide; host: humans; epitope source: viruses; assay: T cell- or B cell-positive; and disease: infectious or autoimmune disease. Hence, we collected 4 types of virus-specific epitopes that were arranged in 4 independent datasets: (A) B cell epitopes identified in the context of autoimmune diseases, (B) B cell epitopes identified in the context of infectious diseases, outside of an autoimmune context; (C) T cell epitopes identified in the context of autoimmune diseases; and (D) T cell epitopes identified in the context of infectious diseases, outside of an autoimmune context. For each epitope, the following data was retrieved from IEDB: epitope ID, sequence, antigen name, antigen accession, virus name, and taxa ID. In addition, for T cell epitopes, we collected the experimental HLA restriction annotated in the IEDB assay data. Epitope datasets were subsequently processed to remove epitopes from human endogenous retroviruses (taxid: 11827) with fewer than 8 residues or more than 25 residues. Likewise, we reduced epitope sequence redundancy in the datasets using CD-HIT [61], keeping only epitopes with unique sequences sharing less than 90% identity.

### 4.2. Generation of Non-Epitopes from Viruses

First, the proteomes in FASTA format of all the distinct viruses encompassing B or T cell epitopes linked to autoimmunity were obtained from UNIPROT after the relevant taxonomic identifiers (taxid) using the following Rest API command: curl “https://rest.uniprot.org/uniprotkb/stream?compressed=false&format=fasta&query=reviewed=true+AND+organism_id=taxid, accessed on 19 June 2025” > taxid.fa. Subsequently, for each B or T cell epitope sequence, a random peptide with the same length as the epitope was picked from the corresponding FASTA file using a PYTHON script. Non-T cell and non-B cell epitopes were generated independently and kept in independent datasets.

### 4.3. Determining Sequence Similarity to Human Antigens

Sequence similarity searches against the human proteome were carried online at the NCBI BLAST site (https://blast.ncbi.nlm.nih.gov/Blast.cgi) (accessed on 15 January 2025), selecting BLASTP [62]. The amino acid sequences of distinct epitope datasets were entered independently as queries in FASTA format, selecting SWISSPROT as the target database and restricting the search to fumans (taxid: 9606). BLASTP searches were carried out with default settings, but the expect value threshold that was set to 10,000. The raw output from the searches was downloaded as text and processed, assigning the maximum bit-score reached with a human protein to each epitope/peptide sequence (B cell epitopes, T cell epitopes, and non-epitopes) and also recording the name of the human protein and its accession. The BLAST bit-score measures sequence similarity independent of query sequence length and database size and is normalized based on the raw pairwise alignment score (S). The bit-score was determined as indicated in [62] by the following formula: bit-score = (λ × S − lnK)/ln2, where λ is the Gumble distribution constant and K is a constant associated with the scoring matrix used, BLOSUM62 in our case. The bit-score and raw alignment score are linearly related, and the higher they are, the more highly significant the match/alignment is. The raw score, S, was calculated by adding the BLOSUM2 scores matching the alignment and subtracting the penalties for opening and extending gaps, determined by the default values set by the NCBI BLAST site.

### 4.4. Other Procedures

Mann–Whitney U tests were performed in order to detect statistically significant differences between the bit-scores of different sets of epitopes, using Holm–Bonferroni corrections for multiple comparisons [63]. Gene enrichment analysis on human proteins with similarity to viral epitopes was carried out as follows. UNIPROT accession numbers of human proteins were converted into gene names using the ID mapping resource at https://www.uniprot.org/id-mapping (accessed on 3 March 2025). The genes associated with these protein records were then utilized to conduct gene set enrichment analysis based on Gene Ontology (GO) terms related to cellular component annotations. Significantly enriched GO terms were identified using the hypergeometric test, adjusting for multiple comparisons using the False Discovery Rate (FDR). GO terms with an adjusted *p*-value ≤ 0.05 were considered statistically significant. Gene enrichment and statistical analyses were performed in RStudio (version 2025.05.0+496), using the clusterProfiler package (version 3.21) [64] and Bioconductor dependency libraries (version 3.21) [65].

## 5. Conclusions

Our work supports a major role of molecular mimicry in triggering/sustaining autoimmunity linked to viral infections, uncovering unnoted drivers of autoimmunity, such as different human coronaviruses and flaviviruses, and anticipating relevant clinical manifestations. It also supports that T cell epitope immunogenicity correlates with similarity rather than dissimilarity to self-antigens, challenging the common view on self/non-self-discrimination for T cells. One limitation of our study is that we identified molecular mimicry solely at the sequence level. This approach is suitable for linear B cell epitopes and T cell epitopes. However, it is not applicable to conformational B cell epitopes, which were not included in this study. The generalizability of our findings and conclusions are limited by potential biases and errors in the immune epitope database (IEDB). The IEDB collects experimentally determined epitopes from published research, resulting in data that is skewed towards the specific interests of researchers in viruses, diseases and HLAs.

## Data Availability

Data are contained within the article and Supplementary Materials.

## Supplementary Materials

The following supporting information can be downloaded at https://www.mdpi.com/article/10.3390/ijms26136041/s1.

## Abbreviations

The following abbreviations are used in this manuscript:

BCR	B Cell Receptor
BLAST	Basic Local Alignment Search Tool
ccHCoVs	Common Cold Human Coronaviruses
DENV	Dengue Virus
FDR	False Discovery Rate
GO	Gene Ontology
HBV	Hepatitis B Virus
HCV	Hepatitis C Virus
HLA	Human Leukocyte Antigen
HPV	Human Papillomavirus
IAV	Influenza A Virus
IEDB	Immune Epitope Database
MERS-CoV	Middle East Respiratory Syndrome Coronavirus
MHC	Major Histocompatibility Complex
NCBI	National Center for Biotechnology Information
SARS-CoV-2	Severe Acute Respiratory Syndrome Coronavirus 2
TCR	T Cell Receptor
TTV	Torque Teno Virus
WNV	West Nile Virus
ZKV	Zika Virus
